# Development of a QPatch-Automated Electrophysiology Assay for Identifying TMEM16A Small-Molecule Inhibitors

**DOI:** 10.1089/adt.2019.962

**Published:** 2020-04-17

**Authors:** Kathryn A. Henckels, David Fong, Jonathan E. Phillips

**Affiliations:** ^1^ Department of Process Development, Amgen, Inc., Thousand Oaks, California, USA.; ^2^ Department of Inflammation Discovery Research, Amgen, Inc., Thousand Oaks, California, USA.

**Keywords:** TMEM16A, small molecule, QPatch, drug development, electrophysiology, ANO1

## Abstract

The calcium-activated chloride channel, TMEM16A, is involved in airway hydration and bronchoconstriction and is a promising target for respiratory disease. Drug development efforts around channels require an electrophysiology-based assay for identifying inhibitors or activators. TMEM16A has proven to be a difficult channel to record on automated electrophysiology platforms due to its propensity for rundown. We developed an automated, whole-cell, electrophysiology assay on the QPatch-48 to evaluate small-molecule inhibitors of TMEM16A. In this assay, currents remained stable for a duration of roughly 11 min, allowing for the cumulative addition of five concentrations of compounds and resulted in reproducible IC_50_s. The absence of rundown was likely due to a low internal free-calcium level of 250 nM, which was high enough to produce large currents, but also maintained the voltage dependence of the channel. Current amplitude averaged 6 nA using the single-hole QPlate and the channel maintained outward rectification throughout the recording. Known TMEM16A inhibitors were tested and their IC_50_s aligned with those reported in the literature using manual patch-clamp. Once established, this assay was used to validate novel TMEM16A inhibitors that were identified in our high-throughput fluorescent-based assay, as well as to assist in structure–activity relationship efforts by the chemists. Overall, we demonstrate an easy to operate, reproducible, automated electrophysiology assay using the QPatch-48 for TMEM16A drug development efforts.

## Introduction

TMEM16A, also referred to as Ano1, is a calcium-activated chloride channel^1–3^ found in the vascular smooth muscle cells of the brain,^4^ the interstitial cells of Cajal in the gut,^5^ and in the respiratory system. In the lung, TMEM16A is expressed in airway epithelial cells, contributing to airway hydration,^6^ and in airway smooth muscle where it controls bronchodilation.^7^ Under normal physiological conditions, TMEM16A displays outwardly rectifying currents with a slow deactivating tail current.^8^

The structure of human TMEM16A was recently solved,^9^ confirming that TMEM16A has 10 transmembrane segments and dimerizes to form a functional channel with 2 independent pores.^10,11^ Two calcium binding sites exist in the pore of each channel,^11^ allowing a total of four calcium ions to bind to a functional channel. TMEM16A is gated in a unique way, through which calcium binds directly to the pore locking the channel in an open state. In a low-calcium environment, the channel opens in response to membrane depolarization.^12^ However, when internal calcium levels are high, reaching above 1 μM free-calcium, the channel loses its voltage dependence and opens in response to the high calcium level. This high-calcium environment leads to a loss of outward rectification, forcing the channel into an open state and leading to current rundown.^9^

TMEM16A has multiple isoforms, with splice variants that may contain segments *a*, *b*, *c*, and *d*.^13^ The different splice variants vary in their expression patterns and have slightly different kinetics and inactivation profiles.^14^ The splice segments confer a different functional significance, for example, the “b” segment on the N-terminus has been shown to bind to calmodulin^15^ and cause a decrease in calcium sensitivity.^13^ These different TMEM16A isoforms can form hetero- or homodimers.^16^

TMEM16A has been found to be overexpressed in many cancer types,^14,17^ and its overexpression is associated with a poorer prognosis.^18,19^ TMEM16A has high potential as a target for various types of cancer^20^ and many TMEM16A small-molecule inhibitors have been found to suppress cell migration and proliferation in cancer cell lines.^21–23^ In addition, a micro-RNA targeting TMEM16A has recently been shown to act as a tumor suppressor in gastric cancer cells,^24^ presenting a new therapeutic modality with respect to inhibiting TMEM16A. Polycystic kidney disease has also been linked to TMEM16A, and TMEM16A inhibitors have been found to reduce the growth of renal cysts *in vitro*.^25^ TMEM16A is also a high-potential target for respiratory disease, including cystic fibrosis, chronic obstructive pulmonary disease (COPD), and asthma. TMEM16A is involved in bronchodilation^7^ where it contributes to airway smooth muscle contraction. More recently, we identified anthelmintics niclosamide, nitazoxanide, and related compounds as potent TMEM16A antagonists that blocked airway smooth muscle depolarization and contraction.^26,27^ Benzbromarone, a TMEM16A inhibitor used as the main reference compound in the development of this assay, relaxed airways and hyperpolarized airway smooth muscle cells.^28^

TMEM16A's role in smooth muscle contraction in combination with its involvement in mucus hydration points to TMEM16A as a potential asthma target. In regard to COPD and cystic fibrosis, TMEM16A activators have been suggested to aid in mucociliary clearance.^29^ Moreover, it was recently shown that TMEM16A is essential for cystic fibrosis transmembrane conductance regulator (CFTR) activation and membrane expression.^30^ In contrast, recent mouse knockout data suggest that the absence of TMEM16A results in defective basal mucus secretion, as well as attenuates the release of proinflammatory mediators.^31,32^ Whether it is more beneficial activating TMEM16A to improve airway hydration/mucociliary clearance or blocking TMEM16A to inhibit smooth muscle membrane depolarization/bronchoconstriction and reduce inflammation in the airways of CF and COPD patients remains to be determined. Finally, TMEM16A is highly expressed in dorsal root ganglion neurons and a contributor to the pain pathway,^17^ as well as being directly activated by noxious heat,^33^ posing a new pain target for drug development. Therefore, TMEM16A continues to be a therapeutically attractive target for a variety of indications.

Currently, there are no specific inhibitors to TMEM16A that have been transferrable to *in vivo* models. Benzbromarone, a potent TMEM16A inhibitor, also inhibits the CFTR and the epithelial sodium channel.^6^ Niflumic acid is a nonspecific inhibitor, targeting many other chloride channels, including glycine receptor channels.^34^ Ani9 and 10aa, the recently reported and most potent TMEM16A inhibitors, were found to be metabolically unstable.^35^ Therefore, efforts to find a TMEM16A inhibitor lacking off-target effects that is metabolically stable and nontoxic *in vivo* are ongoing. Another requirement for TMEM16A to be a viable drug target is for high-throughput assays to be established to screen chemical libraries and validate any findings.

A fluorescence-based eYFP-quench assay has been established for TMEM16A,^6^ and while this serves the purpose for a first-pass high-throughput screen, in our experience, this screen did not identify all TMEM16A full-blockers. 1PBC, a known potent TMEM16A inhibitor,^36^ only caused 40% inhibition in this assay. This was possibly due to the eYFP assay being iodide based, since it has been reported that the anion passing through the pore has an effect on the open state of the channel.^12^ It is also impossible to control the intracellular calcium level in this assay, which could explain the discrepancy in potency.^37^ Moreover, this eYFP assay does not account for compounds that may trigger internal calcium release, thus activating the channel. There is clearly a need for an automated electrophysiology assay for TMEM16A, whether looking at activators or inhibitors. TMEM16A has proven to be a difficult channel for electrophysiology, owing to its fast rundown, small currents, and the fact that it is a ligand-gated channel. In addition, fluoride, typically used in automated patch-clamp assays to improve seal quality, is known to decrease calcium salt solubility. Therefore, a fluoride-free internal solution is preferable when trying to control for a precise internal calcium concentration.

Here we report the development of a QPatch whole-cell electrophysiology screen for the identification of TMEM16A inhibitors and structure–activity relationship (SAR) development efforts. This low-throughput assay can provide concentration–response curves for roughly 100 compounds per week. Optimization of this assay resulted in high-quality seals, stable currents with little rundown, an average of 6 nA peak current amplitude, and maintenance of outward rectification throughout the duration of the assay.

## Materials and Methods

### Cell Line

HEK293T cells stably expressing the human ANO1 channel (isoform acd) were obtained from Scottish Biomedical. Cells were cultured in Sigma Minimum Essential Media containing 10% heat-inactivated fetal bovine serum, 1% penicillin/streptomycin, 1% glutamine, and 600 ng/mL geneticin. Cells were maintained in a 37°C, 5% CO_2_ environment. Cells were passaged every 3 days after they had reached ∼70% confluency and were not allowed to reach a density greater than 1–2 × 10^5^ cells/cm^2^ during routine culture. When subculturing, cells were rinsed once with room temperature 1 × phosphate-buffered saline (PBS; Ca^2+^/Mg^2+^ free), lifted with TrypLE Express, resuspended in prewarmed growth media, and counted using a hemocytometer. Cells were then plated in T150 flasks at a density of 2.9 × 10^4^ cells/cm^2^ to be either used in the assay or subcultured 72 h later.

### Cell Preparation

On the day of the experiment, cells plated at a density of 2.9 × 10^4^ cells/cm^2^ 72 h prior should be ∼70%–80% confluent. After cells were rinsed with prewarmed 1 × PBS (Ca^2+^/Mg^2+^ free), 3 mL of room temperature Detachin solution (Genlantis) was added to the flask and tilted gently two to three times to cover all the cells. Approximately 2 mL of Detachin was aspirated from the flask, leaving 1 mL on the cells, and then placed in the 37°C incubator for 5 min. Once cells had rounded up, the cells were dislodged by tapping the flask gently. The cells were then resuspended in 5 mL of warm serum-free media (EX-CELL ACF CHO Medium; Sigma; supplemented with 4 mM l-glutamine and 10 mM HEPES). A glass Pasteur pipette with a rubber bulb was then used to gently dissociate the cells from one another. Detachin will often leave cells as doublets or clumps, conditions that will disrupt seal formation on the QPatch. The cells were triturated ∼30 times and then, still using the Pasteur pipette, transferred into the QPatch Cell Hotel. This ensures that cells detach from one another leaving a suspension of single cells. A QStirrer was added to the Cell Hotel and the cell suspension was immediately placed on the QPatch device. Cells were utilized within 15 min.

### Test Compounds

Compounds were reconstituted in dimethyl sulfoxide (DMSO; Sigma) as 10 mM stock solutions. An automated dilution machine set up the microtiter plate (MTP ) by making 1:5 dilutions from a starting concentration of 30 μM. The concentration of DMSO was equal for all concentrations tested. Each MTP contained one row of a negative control (DMSO) and a positive control (benzbromarone). A total of eight compounds were run at a time. All compounds tested were produced in-house.

### Electrophysiology

Whole-cell patch-clamp experiments were performed on a QPatch-48-automated electrophysiology platform (Sophion Biosciences) using the 48-channel, single-hole QPlate (resistance 2.0 ± 0.4 MΩ). The external buffer (in mM) was 140 NaCl, 4 KCl, 2 CaCl_2_, 1 MgCl_2_, 10 HEPES, 10 glucose, pH 7.4 (osmolarity = 300), and the internal buffer (in mM) was 110 CsCl, 20 TEA-Cl, 5.374 CaCl_2_, 1.75 MgCl_2_, 10 EGTA, 10 HEPES, 4 Na_2_ATP, pH 7.2 (osmolarity = 310). Cell positioning was set to a 5-s wait time with a positioning pressure of −60 mbar and a positioning time-out of 60 s. Resistance was set to increase 750% to ensure the success of positioning. To obtain a seal, pressure was increased to −70 mbar with an allowed pressure of −130 to −20 mbar and a holding pressure of −20 mbar. The holding potential was set to −70 mV and a 240-s time-out was set. The minimum seal resistance was set to 0.02 GΩ. To go whole cell, five 1-s suction pulses were applied starting at −180 mbar and increasing with −40 mbar increments with a pulse period of 10 s. Suction ramp and voltage zap protocols were kept standard, according to the default HEK cell settings. Voltage zaps had an order of use of 3, ten 4 ms voltage zaps with an amplitude of −400 mV for a zap period of 20 s. The suction ramp order of use was set to 2 with a ramp amplitude of −450 mbar and a ramp slope of 100 ms/mbar.

Once established in whole-cell configuration, cells are clamped to a holding potential (V_hold_) of 0 mV. A standardized IV protocol was used to elicit ionic current through the TMEM16A chloride channel at 20-s intervals. Steady-state voltage pulses begin at −100 to +100 mV in +20 mV steps for a duration of 500 ms. After each pulse, the voltage returns to −100 mV for 50 ms to obtain tail currents, and then returns to the holding potential of 0 mV until the beginning of the next sweep. Block of TMEM16A chloride current due to test article is measured at the +100 mV depolarization sweep of the third and final IV protocol run per concentration addition. This ensures a minimum of 60 s per concentration incubation before measuring current block. Currents are acquired at 10 kHz and filtered at 2 kHz, leak subtraction and R_series_ are disabled, and a 1 MΩ minimum resistance is set. A cumulative concentration response is measured through which each cell is exposed to five concentrations of test article with a dilution factor of 1:5 (*e.g.,* 0.048, 0.24, 1.2, 6, and 30 μM). Cells are recorded for ∼60 s per solution per compound addition. Initially, external solution is applied twice to allow currents to stabilize. Vehicle from the saline reservoir is then added twice, for a total incubation time of 2 min, to monitor any effect 0.3% DMSO might have on currents. This leads directly into the concentration runs by applying Concentration 1 (single addition) for 1 min. Concentration 2 is then applied for the subsequent minute, and so on. Recovery/washout is monitored for a final minute with an external solution addition.

### Data Analysis

The QPatch software automatically generates sweep traces, current time (IT) plots, and IC_50_s when the user opens the data using a predefined template. While there are automatic filters embedded in the template, the user must still inspect the data to ensure the quality of the recordings. Each individual set of traces or trial must be visually inspected and either accepted or rejected. The general criteria for acceptance are as follows:

1. Outward rectification must be present in the first saline addition with inward current smaller than −2 nA.
2. Initial sustained current must be >1 nA.
3. Measured currents must achieve steady state (does not decrement by >10%) before the first compound addition.

If a trial fails to meet one or more of these criteria, then it will be removed from analysis. An automatic filter was enabled through the QPatch software that discards any cells with peak current amplitude measured at +100 mV that is less than 1 nA. An additional filter was set to automatically discard cells that lost outward rectification [I_(+100mV)_/I_(−100mV)_ ≤ 2].

The template selects the peak current at the end of each voltage step by placing the cursor at 540 ms through 549.8 ms of the IV step. Peak outward current magnitude is measured for each sweep at 10-s intervals and is used to generate the IT plot. For further statistics, the IT plot data window captures the peak current at the last +100 mV pulse of each liquid period. Each cell's peak current is then normalized to itself at the initial 0.3% vehicle control period before compound application. This step is to account for differences in current size for each cell. Normalized responses to test article are plotted against their concentrations to reveal a concentration-inhibition plot. A Normalized Group Hill fit is then performed on the plotted results to yield a pooled IC_50_ value, reconstructed from a minimum of two cells per concentration. The baseline response and the full response are constrained to 1 and 0, respectively.

## Results

### Calcium Response

TMEM16A is a calcium-activated channel that is also voltage dependent. It has recently been reported that there are multiple open confirmations, one that is stimulated with high internal calcium levels and another that opens in the presence of low calcium and depolarized membrane potentials.^9^ Which form is physiologically relevant or more important to consider for drug development? Surely there can be internal calcium releases that produce a brief and localized calcium concentration in excess of 1 μM.^38^ However, the channel in the continued presence of high internal calcium levels leads to current rundown, which exaggerates any inhibition caused by a compound. In addition, it is not physiological to have sustained internal calcium levels at such a high concentration. Under resting conditions, the internal calcium level in airway smooth muscle cells is ∼200 nM.^39^ For an assay assessing TMEM16A current, it is ideal to have internal calcium levels be high enough to activate the channel under depolarizing conditions, but not so high as to cause current rundown.

**Fig. 1.**
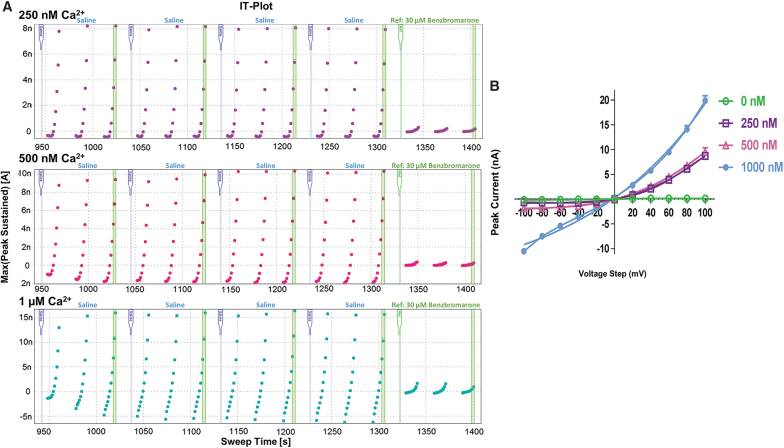
**(A)** Current time (IT) plots with varying concentrations of internal free-calcium. *Top:* 250 nM free internal calcium resulting in stable currents and tightly maintained outward rectification. *Middle:* 500 nM free internal calcium resulting in stable currents, but with a slight leak. *Bottom:* 1 μM free internal calcium resulting in a loss of outwardly rectifying current and leakier current observed in the presence of full blocker benzbromarone. **(B)** Current voltage (IV) plots of TMEM16A currents at different internal free-calcium concentrations; 0 nM (*green*), 250 nM (*purple*), 500 nA (pink), 1 μM (*blue*).

To determine the optimal internal calcium concentration, the multiple ringer reservoir was used and internal solutions with four different free-calcium concentrations were tested: 0, 250, 500, and 1,000 nM. Osmolarity and pH were identical for each of the solutions. As expected, no currents were elicited with the calcium free internal solution at any voltage tested. Current amplitude increased with increasing levels of free-calcium, however, voltage dependence changed. At 250 nM free-calcium level, the current was outwardly rectifying and stable for the duration of the assay (*Fig. 1A*). While peak current amplitude only reached an average of 7 nA, that is more than sufficient for identifying inhibitors. The 500 nM free-calcium condition also produced acceptable results, however, there was a slight leak observed with the −100 mV pulse. Furthermore, the currents were not as stable toward the end of the assay when compared with the 250 nM condition. The condition with 1 μM free-calcium, while producing a large current of 20 nA also caused the channel to lose outward rectification and resulted in an almost linear IV curve (*Fig. 1B*). It was decided that using a lower, more physiologically relevant calcium level is preferable to maintain stable currents and outward rectification.

### Establishing the Assay: Solving for Current Stability

If the cells are prepared in accordance with the protocol, seals were of regularly high quality, with initial seals over 1 GΩ. Throughout the recording, minimum seal resistance was kept at 1 MΩ due to some of the channels being in an open state and thereby lowering the resistance. Nevertheless, seal quality can be assessed by monitoring whether a cell maintains outward rectification throughout the recording. Cells exhibiting inward current, with IT plots resembling the 1 μM internal free-calcium condition, were found to have lost resistance and seal quality. Presumably, loss of seal resistance can lead to a “leaky” cell, allowing an influx of calcium from the external environment. This can mask the inhibitory effects of a compound since it was found that cells with low seal resistance tend to display residual current with inhibitors that are normally full blockers. This skews results toward a higher IC_50_ and causes much greater variability in the data when grouped together with high-quality cells that received the same compound. In addition, if internal calcium levels increase substantially, current will exhibit rundown, which will result in a lower IC_50_, reporting an increased potency for the compound. Again, when grouped together with cells with high-quality recordings that received the same compound, the standard deviation (SD) will be high leading to a wide 95% confidence interval. Therefore, it is important to identify and remove these cells from further analysis. Filters were set so that any cell meeting the criteria of I_(+100)_/I_(−100)_ ≤ 2 or I_(−100)_ ≤ −1 nA was automatically excluded.

**Fig. 2.**
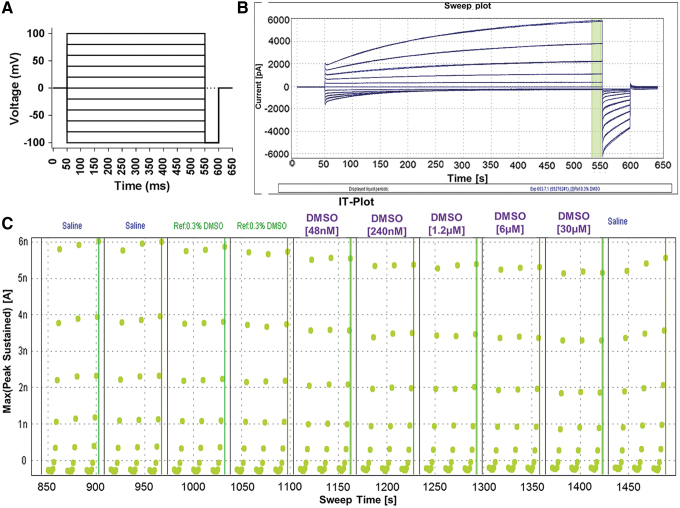
**(A)** QPatch assay voltage protocol. Protocol consists of +20 mV steps from −100 mV to +100 mV that are 500 ms long from a 0 mV holding potential ending in a 50 ms −100 mV pulse to monitor deactivation before returning to a holding potential of 0 mV until the start of the next step. **(B)** Typical sweep plot of TMEM16A current on QPatch during one IV step protocol after a saline (external solution) addition. The highlighted *green bar* represents area of peak current used to generate the IT plot. **(C)** IT plot with liquid additions of 0.3% DMSO from MTP. Current remains stable, with a peak current amplitude of 6 nA at the +100 mV pulse. Outward rectification is observed by the lack of inward current (< −1 nA). DMSO, dimethyl sulfoxide; MTP, microtiter plate.

To be able to constantly monitor the outward rectification, a voltage step protocol was designed. Cells were held at 0 mV holding potential and then 500 ms pulses were applied from −100 to +100 mV in +20 mV steps. Cells were then pulsed at −100 mV for 50 ms to monitor deactivation before returning to the holding potential of 0 mV until the voltage protocol repeated (*Fig. 2A*). The voltage protocol is set to allow 10 s from beginning to end, and the cells are held at 0 mV until the next voltage protocol is initiated. A ramp protocol was tried initially; however, it tended to cause greater rundown and did not provide output that was as user friendly to quality control as the voltage step protocol. A 0 mV holding potential was chosen because it allowed for the most stable currents throughout the assay. Holding potentials of −70 and −40 mV were tried, however, they caused an average of 28% and 25% rundown, respectively. The 0 mV holding potential is also the reversal potential for the Cl^−^ in this assay, so if there are some open channels at this potential, there will be no net flux of ions, providing for a clean readout in the sweep and IT plots. This voltage protocol resulted in stable currents that increased in amplitude with increasing depolarization and had little to no inward current (*Fig. 2B*). Seals were over 1 GΩ and resistance typically dropped to 300–500 MΩ after whole cell was achieved. Data were excluded from cells that dropped below 100 MΩ by the end of the protocol.

The peak current from the last 10 ms of each voltage step was used to generate the IT plot (*Fig. 2C*). The IT plot displays three voltage protocols from each liquid period. The last current recorded at +100 mV for each liquid period is used for further data analysis, statistics, and to generate an IC_50_.

It is ideal to have at least 8 concentrations to fit an IC_50_ curve and for most high-throughput electrophysiology assays, a 22-point curve is standard. However, TMEM16A is a difficult channel to record from for extended periods. The channel is prone to rundown, and current stability decreases as the recording time increases. Evaluating eight compound concentrations caused the assay to run too long, and the control cells receiving 0.3% DMSO began to exhibit rundown. After attempting many different recording lengths ranging from 5 to 20 min, a roughly 11-min assay was established comprising five compound additions (*Table 1*). Using a 1:5 dilution, the 5-point inhibition curves generated reproducible IC_50_s that aligned with those reported in the literature. The application protocol calls for two saline addition periods to stabilize the current and obtain baseline currents. Then, two 60-s liquid periods with additions from the reference reservoir are to control for any affect the vehicle, in this case 0.3% DMSO, has on current. Next, the lowest dose of compound is added from the MTP and three consecutive voltage protocols are run resulting in a 1-min incubation time for that compound concentration. Following that liquid period, the next highest dose is added to the cell from the MTP. This continues until all five concentrations have been added, each with an incubation time of 1 min, resulting in a total compound incubation time of ∼5 min. “Approximately” is used because the QPatch can vary slightly in timing, depending on the movement of the pipettes, the number of cells patched, and the number of compounds on the MTP. The assay concludes with a saline addition to look for washout effects.

**Table 1.** Application Protocol

Liquid period	Liquid	Volume (μL)	VP runs
1	Saline	5	3
2	Saline	5	3
3	Vehicle	5	3
4	Vehicle	5	3
5	Concentration 1	5	3
6	Concentration 2	5	3
7	Concentration 3	5	3
8	Concentration 4	5	3
9	Concentration 5	5	3
10	Saline	5	3

VP, voltage potential.

**Fig. 3.**
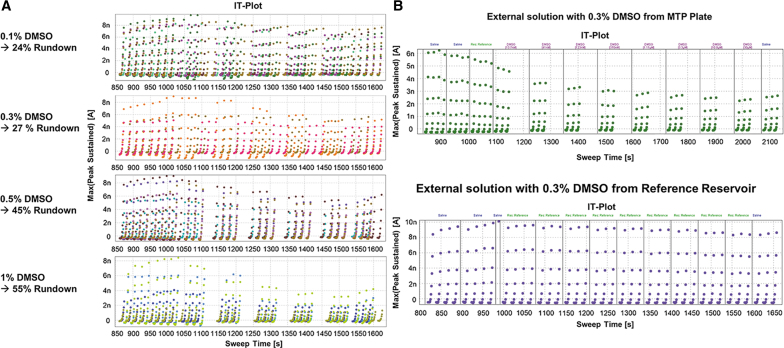
**(A)** Current time plots with varying concentrations of DMSO. *Top:* 0.1% DMSO resulting in 24% rundown at the end of the assay. *Middle top*: 0.3% DMSO resulting in 27% rundown. *Middle bottom*: 0.5% DMSO resulting in 45% rundown. *Bottom:* 1% DMSO resulting in 55% rundown at the end of the assay. **(B)** Current time plots showing differences in rundown with 0.3% DMSO depending on where liquid is added from. *Top:* Liquid added to QPlate from MTP. *Bottom:* Liquid added to QPlate from reservoir.

DMSO tolerance was assessed and it was determined that 0.1% and 0.3% DMSO had similar effects on channel current, whereas DMSO concentrations greater than 0.5% caused increased current rundown (*Fig. 3A*). A 0.3% DMSO concentration was chosen for all compounds, since this allowed for higher concentrations to be tested when diluted from a 10 mM stock solution. The effect of 0.3% DMSO on the cells was initially tested by applying external solution containing 0.3% DMSO from the MTP. This led to an average of 27% rundown, which was considered acceptable for this particular channel. However, a strange phenomenon was discovered when 0.3% DMSO was added from the vehicle reservoir instead of the MTP. In this case, there was zero rundown and currents remained perfectly stable throughout a 14-min assay (*Fig. 3B*). Therefore, 0.3% DMSO has no effect on current, and the rundown that was observed was due to how the machine was adding liquid to the QPlate.

A time delay is noticeable between liquid periods when liquid is taken from the MTP. This is due to the ability of the pipettes to move to the MTP, aspirate liquid, move back to the QPlate and deliver the liquid to the appropriate wells, rinse the pipette tips, and return to the MTP for the next concentration of compound in a timely manner. If the pipettes cannot return to the QPlate in time to deliver the next concentration of compound, meaning as soon as the third voltage protocol has concluded, the cell will remain at the holding potential in the previously delivered liquid. This delay in the assay is causing current rundown. When the pipettes only have to move from the vehicle reservoir and back to the QPlate, without washing the pipettes or minding which cell receives which compound, there is no lag time and no rundown (*Fig. 3B*). To decrease this lag time between liquid periods, the protocol was modified to run six voltage protocols per liquid period. During the delay between compound additions, cells were held at 0 mV potential. However, there was still a lag time present even when doubling the voltage protocol runs, and rundown persisted. Extending the assay further would cause the total assay time to be too long, and therefore, another way to mitigate this lag time between liquid periods would have to be implemented.

**Fig. 4.**
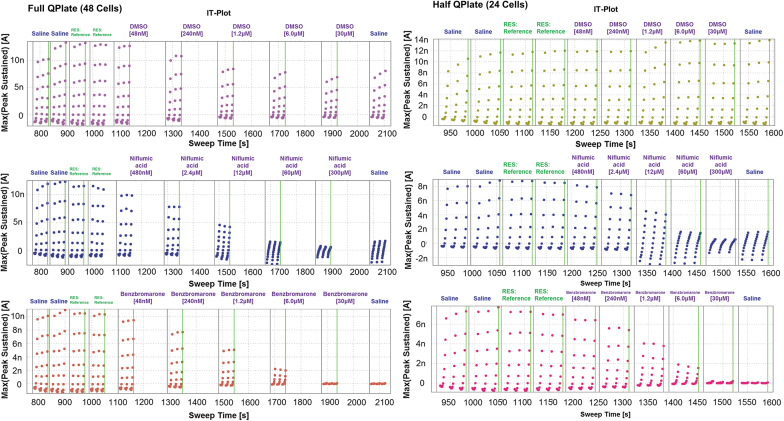
Current time plots contrasting running a full QPlate (*left*) with a half QPlate (*right*) adding compounds from the MTP. *Top:* 0.3% DMSO, *middle:* niflumic acid, *bottom:* benzbromarone.

One feature of the QPatch-48 is that you can choose which columns on the QPlate you would like to use in an experiment. You can select just one, for instance column A, and run the experiment with a maximum of eight cells. In addition, you can choose to run half the plate for a total of 24. When the assay was run as a half plate, the time lag was alleviated and there was no longer rundown present in the cells receiving 0.3% DMSO (*Fig. 4*). The inhibition curves for known TMEM16A inhibitors also became more consistent when the assay was run using a half 24-well format. The rundown from the time lag between liquid periods exaggerated the inhibition caused by the compound, overestimating its potency. When benzbromarone was run using a full 48-well plate format, the IC_50_ reported was 730 nM (*n* = 1). However, when run with the half plate, the average IC_50_ was 2.54 ± 0.94 μM (mean ± SD, *n* = 5). The same effect was seen with the niflumic acid, where an IC_50_ of 3.89 ± 0.11 μM (*n* = 3) was reported with the full plate and a rundown-free half plate generated an IC_50_ of 8.34 ± 3.77 μM (*n* = 5). Fortunately, the “Rolling Plate” setting in the QPatch software allowed the assay to run on the half plate, and once finished, plated cells from the cell hotel into the other half of the plate to continue the assay. This did extend the time required to run 48 cells, but not by a significant amount, because there was no lag time while the assay was being run. It also required no further user intervention, as the machine automatically loaded the second half of the plate with cells and solutions once the first half of the plate was finished. This minor change in the flow of the assay greatly increased the quality of the data.

### Obtaining Dose Responses Using Benchmark Small-Molecule Inhibitors

**Fig. 5.**
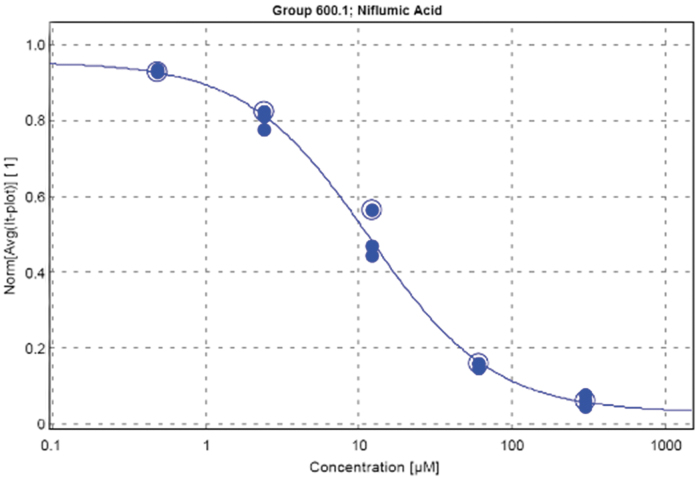
Dose–response curve for niflumic acid (*n* = 3) resulting in a calculated IC_50_ of 11.12 μM.

To determine whether the QPatch assay can identify TMEM16A small-molecule inhibitors, two benchmark inhibitors were chosen to validate the assay. Benzbromarone^6^ and niflumic acid, two established TMEM16A inhibitors, were chosen to validate that the assay generated expected dose–response curves. Niflumic acid repeatedly produced an IC_50_ of 8.34 ± 3.77 μM (*n* = 5), aligning nicely with the reported IC_50_ of 12 μM.^8^ In addition, niflumic acid resulted in a slower deactivation rate, which is typical for this compound.^8^ Benzbromarone resulted in IC_50_ values slightly more potent than those reported in the literature, but still within a threefold window. Besides producing expected IC_50_ values in this assay, these IC_50_s were extremely reproducible over multiple runs, whether the assay was performed on the same day or on different days (*Table 2*). Due to the time constraints on the assay, a 1:5 dilution was determined to generate the best IC_50_ curves. The dose–response curve generated by the QPatch software for niflumic acid exemplifies how little variability there is between cells within an experiment (*Fig. 5*). Once the assay was established, other known TMEM16A inhibitors were evaluated (*Table 2*). Ani9 and its analogue^40^ were tested, and dose–response curves that were generated produced IC_50_s within the expected range. Furthermore, for those compounds that were run more than once, either on the same or different days, the results were extremely reproducible, further strengthening the validity of the assay. 1PBC, another known TMEM16A inhibitor, resulted in almost 10 times the potency as that reported^36^ in this QPatch assay. This could be due to a variety of factors, including the isoform and species of the channel (Peter *et al.* used mouse TMEM16A) and that the internal calcium concentration has a large effect on the potency of 1PBC, presumably by allowing access to the pore. Unfortunately, this assay was unable to see more than a 40% block with T16Ainh-A01, an aminophenylthiazole known to be a potent blocker of TMEM16A.^41^ This could be due to solubility issues, a slow-onset response, or to the splice variant (TMEM16A abd) being tested. Taken altogether, the reproducibility of the known inhibitors and the resultant IC_50_s validated this assay for hit confirmation and SAR efforts around a small-molecule TMEM16A inhibitor.

**Table 2.** Potency of TMEM16A Inhibitors Generated by QPatch Assay

Name	IC_50_s (μM) for independent experiments				
	1	2	3	4	5
Benzbromarone	3.45	1.51	1.31	2.30	3.18
1PBC	1.18	0.85	1.10	1.57	—
Niflumic acid	5.29	4.62	8.45	11.12	7.71
Niclosamide	1.62	1.43	1.05	2.09	1.81
Ani9	0.067	0.054	0.078	—	—
AMG compound	0.428	0.510	0.258	0.443	0.470

**Fig. 6.**
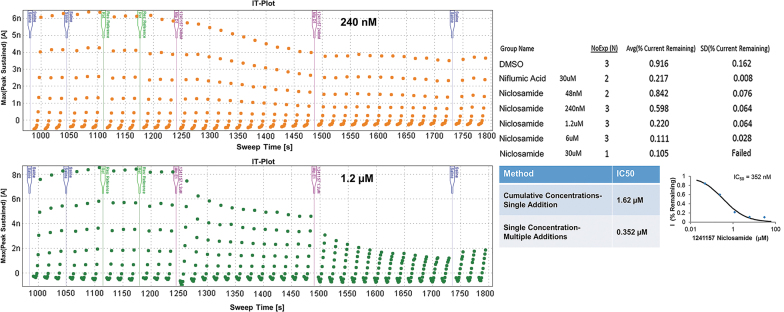
Current time plots of the modified protocol with multiple additions of a single concentration. *Top:* IT plot when 240 nM niclosamide is added to a cell. *Bottom:* IT plot when 1.2 μM niclosamide is added to a cell. *Right:* Dose–response results and curve for niclosamide in the modified single-concentration assay result in an IC_50_ of 352 nM, compared with the cumulative addition protocol IC_50_ of 1.62 μM.

As mentioned, due to the nature of the channel and its rundown issues, time limitations exist for recording. Therefore, each concentration of compound is added to the cell for a duration of 60 s. It is possible that the IC_50_ is shifted for those compounds that require a longer time to act on the channel. To account for this, a single-concentration/multiple-addition protocol was established (*Fig. 6*). We found that niclosamide inhibited 50% of channel current at ∼1 μM on the QPatch, however, in many other in-house assays it was twice as potent.^42^ This led us to wonder if we were missing the full response to the concentration before the next liquid period began and the more concentrated, cumulative addition was applied to the cell. The original application protocol was amended to allow for two 4-min incubations with the same concentration of compound. The total length of the assay is roughly 15 min, with two 1-min saline additions to stabilize current, two 1-min vehicle additions to account for any DMSO effects, and two 4-min compound additions, ending with two 1-min saline additions to look for washout effects. The same IV voltage protocol was used as well as the same whole-cell conditions and solutions. When niclosamide was run with this extended incubation protocol, the IC_50_ was four times as potent (*Fig. 6*), aligning nicely with the other in-house assays that were run. A DMSO condition was included to account for any rundown that occurred, with the result of 92% current remaining at the end of the assay. Therefore, the block observed with niclosamide additions was not due to rundown. When 240 nM was added, the current plateaued at ∼60% current amplitude. The 1.2 μM condition showed a dramatic decrease in current after the first addition that ended with a 50% block in current after the 4-min incubation, but on the second addition, current dropped another 20% for a final response of 78% block. The final IC_50_ was reported as 221 ± 185 nM (*n* = 2), when the single-addition, cumulative concentration assay produced an IC_50_ of 1.60 ± 0.39 μM (*n* = 5). Therefore, this low-throughput, extended assay can be used to determine whether some compounds that are slower acting are more potent than what was being uncovered in the standard 1-min incubation assay. Any compounds that result in partial inhibition with the original assay can be run on the multiple-addition assay to see if they require more time to inhibit the channel.

## Discussion

Using the QPatch-48, we have developed a robust assay for the assessment of TMEM16A small-molecule inhibitors. Many optimization steps were performed to determine the optimal cell culture conditions, internal calcium level, holding potential and voltage protocol, and maximum duration of the assay. It was necessary to control for the culture conditions of the cells and strictly adhere to the cell preparation procedure to obtain high-quality seals. In addition, while the external solution can be used throughout the day, the internal solution should be replaced every 2 h to ensure that the ATP is fresh. Best practices also require solutions to be made weekly and for the osmolarity and pH to be measured accurately. Having met these conditions, the assay results in large currents, averaging a peak current of 6 nA, while maintaining outward rectification that is characteristic of the TMEM16A channel. The benchmark TMEM16A inhibitors that were tested during the validation efforts generated reproducible and expected IC_50_ values. This assay was established using HEK293T cells that stably expressed TMEM16A(acd), however, we found that this assay was easily transferable to other cell lines. Another HEK293T cell line that stably expressed the abc isoform of TMEM16A also formed high-quality seals and produced large currents. The currents remained stable for the duration of the assay and were fully blocked by 30 μM benzbromarone.

In addition, another cell line that was HEK293T-derived and induced expression of TMEM16A on addition of 10 ng/mL of doxycycline also performed well on the assay. Currents averaged 8 nA, were fully blocked by 30 μM benzbromarone, and maintained outward rectification throughout the assay. These two other cell lines were run on the machine using the predefined parameters that were optimized with the original cells, including the cell preparation protocol. Therefore, it seems that this assay is easily transferable to any cell line expressing TMEM16A.

In summary, we developed an automated electrophysiology assay on the QPatch-48 for the assessment of TMEM16A small-molecule inhibitors. Using a low internal free-calcium concentration (250 nM), currents were kept extremely stable throughout the duration of the assay, exhibiting little to no rundown typical of the channel. In addition, a voltage protocol was designed to monitor the continued presence of outward rectification, allowing an automatic quality control filter to be applied to the data. This ensured that internal calcium levels remained low and that data were collected from quality recordings. IC_50_s for known TMEM16A inhibitors aligned with published data and were highly reproducible. For slower acting compounds, an extended protocol assessing the effect of multiple additions of a single concentration was established.

This method is now being extended to evaluate large-molecule candidates with a slightly modified liquid application protocol. The TMEM16A QPatch-48 assay, while only capable of assaying 100 compounds a week, greatly accelerated our TMEM16A small-molecule program by providing clear results on compounds in question. The quick data turnaround time facilitated the medicinal chemistry team in these SAR efforts. It is possible that this assay could be slightly amended for use on the Qube, the 384-well whole-cell machine made by Sophion. The two machines are similar enough that the protocol should be able to be transferred to the Qube for a high-throughput TMEM16A inhibitor screen. This would enable a library screen to be executed with electrophysiology, instead of relying solely on a fluorescent-based screening assay.

## Abbreviations Used

CFTR: cystic fibrosis transmembrane conductance regulator
COPD: chronic obstructive pulmonary disease
DMSO: dimethyl sulfoxide
MTP: microtiter plate
PBS: phosphate-buffered saline
SAR: structure–activity relationship
SD: standard deviation

## References

[1] Caputo A, Caci E, Ferrera L, *et al.*: TMEM16A, a membrane protein associated with calcium-dependent chloride channel activity. Science 2008;322:590–594 18772398 10.1126/science.1163518

[2] Schroeder BC, Cheng T, Jan YN, Jan LY: Expression cloning of TMEM16A as a calcium-activated chloride channel subunit. Cell 2008;134:1019–1029 18805094 10.1016/j.cell.2008.09.003 PMC2651354

[3] Yang YD, Cho H, Koo JY, *et al.*: TMEM16A confers receptor-activated calcium-dependent chloride conductance. Nature 2008;455:1210–1215 18724360 10.1038/nature07313

[4] Du YH, Guan YY: Chloride channels—new targets for the prevention of stroke. Curr Vasc Pharmacol 2015;13:441–448 25360844 10.2174/1570161112666141014145040

[5] Huang F, Rock JR, Harfe BD, *et al.*: Studies on expression and function of the TMEM16A calcium-activated chloride channel. Proc Natl Acad Sci U S A 2009;106:21413–21418 19965375 10.1073/pnas.0911935106 PMC2781737

[6] Huang F, Zhang H, Wu M, *et al.*: Calcium-activated chloride channel TMEM16A modulates mucin secretion and airway smooth muscle contraction. Proc Natl Acad Sci U S A 2012;109:16354–16359 22988107 10.1073/pnas.1214596109 PMC3479591

[7] Zhang CH, Li Y, Zhao W, *et al.*: The transmembrane protein 16A Ca(2+)-activated Cl- channel in airway smooth muscle contributes to airway hyperresponsiveness. Am J Respir Crit Care Med 2013;187:374–381 23239156 10.1164/rccm.201207-1303OC PMC3603598

[8] Bradley E, Fedigan S, Webb T, *et al.*: Pharmacological characterization of TMEM16A currents. Channels (Austin) 2014;8:308–320 24642630 10.4161/chan.28065 PMC4203732

[9] Paulino C, Kalienkova V, Lam AKM, Neldner Y, Dutzler R: Activation mechanism of the calcium-activated chloride channel TMEM16A revealed by cryo-EM. Nature 2017;552:421–425 29236691 10.1038/nature24652

[10] Jeng G, Aggarwal M, Yu WP, Chen TY: Independent activation of distinct pores in dimeric TMEM16A channels. J Gen Physiol 2016;148:393–404 27799319 10.1085/jgp.201611651 PMC5089935

[11] Dang S, Feng S, Tien J, *et al.*: Cryo-EM structures of the TMEM16A calcium-activated chloride channel. Nature 2017;552:426–429 29236684 10.1038/nature25024 PMC5750132

[12] Peters CJ, Gilchrist JM, Tien J, *et al.*: The sixth transmembrane segment is a major gating component of the TMEM16A calcium-activated chloride channel. Neuron 2018;97:1063–1077.e1064. 29478917 10.1016/j.neuron.2018.01.048 PMC5860880

[13] Ferrera L, Caputo A, Ubby I, *et al.*: Regulation of TMEM16A chloride channel properties by alternative splicing. J Biol Chem 2009;284:33360–33368 19819874 10.1074/jbc.M109.046607 PMC2785179

[14] Pedemonte N, Galietta LJ: Structure and function of TMEM16 proteins (anoctamins). Physiol Rev 2014;94:419–459 24692353 10.1152/physrev.00039.2011

[15] Tian Y, Kongsuphol P, Hug M, *et al.*: Calmodulin-dependent activation of the epithelial calcium-dependent chloride channel TMEM16A. FASEB J 2011;25:1058–1068 21115851 10.1096/fj.10-166884

[16] Ohshiro J, Yamamura H, Saeki T, Suzuki Y, Imaizumi Y: The multiple expression of Ca(2)(+)-activated Cl(−) channels via homo- and hetero-dimer formation of TMEM16A splicing variants in murine portal vein. Biochem Biophys Res Commun 2014;443:518–523 24321548 10.1016/j.bbrc.2013.11.117

[17] Oh U, Jung J: Cellular functions of TMEM16/anoctamin. Pflugers Arch 2016;468:443–453 26811235 10.1007/s00424-016-1790-0 PMC4751194

[18] Duvvuri U, Shiwarski DJ, Xiao D, *et al.*: TMEM16A induces MAPK and contributes directly to tumorigenesis and cancer progression. Cancer Res 2012;72:3270–3281 22564524 10.1158/0008-5472.CAN-12-0475-T PMC3694774

[19] Ruiz C, Martins JR, Rudin F, *et al.*: Enhanced expression of ANO1 in head and neck squamous cell carcinoma causes cell migration and correlates with poor prognosis. PLoS One 2012;7:e43265 22912841 10.1371/journal.pone.0043265 PMC3422276

[20] Wang H, Zou L, Ma K, *et al.*: Cell-specific mechanisms of TMEM16A Ca(2+)-activated chloride channel in cancer. Mol Cancer 2017;16:152 28893247 10.1186/s12943-017-0720-x PMC5594453

[21] Mazzone A, Eisenman ST, Strege PR, *et al.*: Inhibition of cell proliferation by a selective inhibitor of the Ca(2+)-activated Cl(−) channel, Ano1. Biochem Biophys Res Commun 2012;427:248–253 22995309 10.1016/j.bbrc.2012.09.022 PMC3479349

[22] Seo Y, Park J, Kim M, *et al.*: Inhibition of ANO1/TMEM16A chloride channel by idebenone and its cytotoxicity to cancer cell lines. PLoS One 2015;10:e0133656 26196390 10.1371/journal.pone.0133656 PMC4511415

[23] Sui Y, Wu F, Lv J, *et al.*: Identification of the novel TMEM16A inhibitor dehydroandrographolide and its anticancer activity on SW620 cells. PLoS One 2015;10:e0144715 26657333 10.1371/journal.pone.0144715 PMC4686118

[24] Cao Q, Liu F, Ji K, *et al.*: MicroRNA-381 inhibits the metastasis of gastric cancer by targeting TMEM16A expression. J Exp Clin Cancer Res 2017;36:29 28193228 10.1186/s13046-017-0499-z PMC5307754

[25] Buchholz B, Faria D, Schley G, Schreiber R, Eckardt KU, Kunzelmann K: Anoctamin 1 induces calcium-activated chloride secretion and proliferation of renal cyst-forming epithelial cells. Kidney Int 2014;85:1058–1067 24152967 10.1038/ki.2013.418

[26] Miner K, Labitzke K, Liu B, *et al.*: Drug repurposing: the anthelmintics niclosamide and nitazoxanide are potent TMEM16A antagonists that fully bronchodilate airways. Frontiers in Pharmacology 2019;10:51 30837866 10.3389/fphar.2019.00051 PMC6382696

[27] Cabrita I, Benedetto R, Schreiber R, Kunzelmann K: Niclosamide repurposed for the treatment of inflammatory airway disease. JCI Insight 2019;4:e128414 10.1172/jci.insight.128414 PMC6693830 31391337

[28] Danielsson J, Perez-Zoghbi J, Bernstein K, et al.: Antagonists of the TMEM16A calcium-activated chloride channel modulate airway smooth muscle tone and intracellular calcium. Anesthesiology 2015;123:569–581 26181339 10.1097/ALN.0000000000000769 PMC4543527

[29] Namkung W, Yao Z, Finkbeiner WE, Verkman AS: Small-molecule activators of TMEM16A, a calcium-activated chloride channel, stimulate epithelial chloride secretion and intestinal contraction. FASEB J 2011;25:4048–4062 21836025 10.1096/fj.11-191627 PMC3205834

[30] Benedetto R, Ousingsawat J, Wanitchakool P, et al.: Epithelial chloride transport by CFTR requires TMEM16A. Sci Rep 2017;7:12397 28963502 10.1038/s41598-017-10910-0 PMC5622110

[31] Benedetto R, Cabrita I, Schreiber R, Kunzelmann K: TMEM16A is indispensable for basal mucus secrestion in airways and intestine. FASEB J 2019;33:4502–4512 30586313 10.1096/fj.201801333RRR

[32] Kunzelmann K, Ousingsawat J, Cabita I, et al.: TMEM16A in cystic fibrosis: activating or inhibiting? Front Pharmacol 2019;10:3 30761000 10.3389/fphar.2019.00003 PMC6362895

[33] Cho H, Yang YD, Lee J, et al.: The calcium-activated chloride channel anoctamin 1 acts as a heat sensor in nociceptive neurons. Nat Neurosci 2012;15:1015–1021 22634729 10.1038/nn.3111

[34] Maleeva G, Peiretti F, Zhorov BS, Bregestovski P: Voltage-dependent inhibition of glycine receptor channels by niflumic acid. Front Mol Neurosci 2017;10:125 28559795 10.3389/fnmol.2017.00125 PMC5432571

[35] Truong EC, Phuan PW, Reggi AL, *et al.*: Substituted 2-acylaminocycloalkylthiophene-3-carboxylic acid arylamides as inhibitors of the calcium-activated chloride channel transmembrane protein 16A (TMEM16A). J Med Chem 2017;60:4626–4635 28493701 10.1021/acs.jmedchem.7b00020 PMC5516794

[36] Peters CJ, Yu H, Tien J, Jan YN, Li M, Jan LY: Four basic residues critical for the ion selectivity and pore blocker sensitivity of TMEM16A calcium-activated chloride channels. Proc Natl Acad Sci U S A 2015;112:3547–3552 25733897 10.1073/pnas.1502291112 PMC4371966

[37] Sung TS, O'Driscoll K, Zheng H, *et al.*: Influence of intracellular Ca2+ and alternative splicing on the pharmacological profile of ANO1 channels. Am J Physiol Cell Physiol 2016;311:C437–C451 27413167 10.1152/ajpcell.00070.2016 PMC5129757

[38] Tadross MR, Tsien RW, Yue DT: Ca2+ channel nanodomains boost local Ca2+ amplitude. Proc Natl Acad Sci U S A 2013;110:15794–15799 24019485 10.1073/pnas.1313898110 PMC3785779

[39] Jude JA, Wylam ME, Walseth TF, Kannan MS: Calcium signaling in airway smooth muscle. Proc Am Thorac Soc 2008;5:15–22 18094080 10.1513/pats.200704-047VS PMC2645299

[40] Seo Y, Lee HK, Park J, *et al.*: Ani9, a novel potent small-molecule ANO1 inhibitor with negligible effect on ANO2. PLoS One 2016;11:e0155771 27219012 10.1371/journal.pone.0155771 PMC4878759

[41] Namkung W, Phuan PW, Verkman AS: TMEM16A inhibitors reveal TMEM16A as a minor component of calcium-activated chloride channel conductance in airway and intestinal epithelial cells. J Biol Chem 2011;286:2365–2374 21084298 10.1074/jbc.M110.175109 PMC3023530

[42] Miner K, Liu B, Wang P, *et al.*: The anthelmintic niclosamide is a potent TMEM16A antagonist that fully bronchodilates airways. bioRxiv 2018; 254888. DOI: 10.1101/254888

